# Chloramine/Chlorine Injury Treated with Noninvasive Positive Pressure Ventilation: A Report of Two Cases

**DOI:** 10.5811/cpcem.43950

**Published:** 2025-09-23

**Authors:** Richard Fisher, Cyrus E. Kuschner, Michael A. Goldstein, Soha Jhaveri, Sanjay Mohan, Payal Sud

**Affiliations:** * Northwell Health, Division of Medical Toxicology, Department of Emergency Medicine, New Hyde Park, New York; † Feinstein Institutes for Medical Research, Northwell Health, New Hyde Park, New York; ‡ Northwell Health, Department of Emergency Medicine, New Hyde Park, New York; § NYU Grossman Long Island School of Medicine, Department of Emergency Medicine, Mineola, New York

**Keywords:** *chlorine*, *chloramine*, *respiratory distress*, *case report*

## Abstract

**Introduction:**

Chlorine and chloramine gases are pulmonary irritants that can cause pulmonary edema and acute respiratory distress syndrome (ARDS). We present two cases that show effective treatment with noninvasive positive pressure ventilation (NIPPV).

**Case Reports:**

**Case 1**. A 9-year-old male developed chloramine pneumonitis and ARDS with hypoxia to 78% on room air after urinating in a bucket of sodium hypochlorite. He was placed on NIPPV with improvement in symptoms and discharged on day four.

**Case 2**. A 58-year-old male developed chlorine gas pneumonitis with hypoxia to 85% on room air. Point-of-care ultrasound of this patient demonstrated greater than three B-lines in bilateral lower lung fields, which resolved after initiating NIPPV. He ultimately left against medical advice.

**Conclusion:**

Noninvasive positive pressure ventilation can be an effective treatment modality for severe lung injury secondary to chlorine or chloramine exposure.

## INTRODUCTION

Chlorine and chloramine gases are common pulmonary irritants that can lead to a spectrum of clinical manifestations, from coughing to bronchospasm to pulmonary edema and acute respiratory distress syndrome (ARDS). The most common setting of exposure is the mixture of household cleaning solutions due to the reaction of sodium hypochlorite (commonly referred to as bleach) with ammonia or a strong acid.^1^ This has been particularly noticeable since the coronavirus disease 2019 pandemic, during which a sharp rise in chlorine and chloramine exposure was noted due to increased disinfectant use.^2^ Historically, severe toxicity leading to fatality has occurred in the context of mass casualty events after chemical warfare or occupational exposures, such as the 2009 train derailment in South Carolina.^3^,^4^ There is also the potential for exposure of chloramine development from a mixture of urine and bleach, given that the ammonia in urine can react with the sodium hypochlorite. While media reports advise not to urinate in a solution of bleach, no documented cases of severe health effects have been reported in the literature to date.

Chlorine gas is known to cause dose-dependent ARDS.^3^,^4^ Initial symptoms after exposure include olfactory and pulmonary effects, such as coughing and nasopharyngeal irritation, with more water-soluble chemicals causing quicker onset of action of symptoms and, thus, more immediate recognition potential to limit severity of exposure.^5^ However, larger exposures to chlorine gas or chemicals with intermediate- or low-water solubility, such as heavier chloramine compounds, are able to penetrate deeper into the respiratory system. This leads to delayed symptom onset, delayed recognition of exposure, and higher severity of presentation.

Treatment of chlorine-based chemical irritants focuses on symptomatic management. For most patients this will be bronchospasm, particularly in patients with reactive airway disease. However, in those with signs of pulmonary edema, further interventions will be needed. To date, there are few reported cases detailing the use of noninvasive positive pressure ventilation (NIPPV) in chlorine gas exposure, with no cases regarding chloramine toxicity.^6^,^7^

We present two cases, the first of which is an uncommon exposure to chloramine. The cases demonstrate that NIPPV can effectively assist in the treatment of chlorine- and chloramine gas-associated pulmonary toxicity.

## CASE REPORTS

### Case 1: Chloramine

A nine-year-old male with no past medical history presented with dyspnea and hypoxia after urinating in a bucket of sodium hypochlorite. Within minutes after the incident, he developed chest discomfort associated with three episodes of emesis. Four hours later he experienced new-onset difficulty breathing, prompting evaluation in the emergency department (ED).

*CPC-EM Capsule*

What do we already know about this clinical entity?

*Chlorine and chloramine gas are pulmonary irritants that can range in severity of disease presentation*.

What makes this presentation of disease reportable?

*Two cases were treated with non-invasive ventilation; one involved an uncommon chloramine exposure after urinating in a bleach-filled bucket*.

What is the major learning point?

*The pathophysiology and management of chlorine and chloramine gas injury, with the potential role of point-of-care ultrasound*.

How might this improve emergency medicine practice?

*Consider non-invasive positive pressure ventilation early for chlorine/chloramine lung injury. Remain cognizant of common household items leading to exposure*.

**Image 1:**
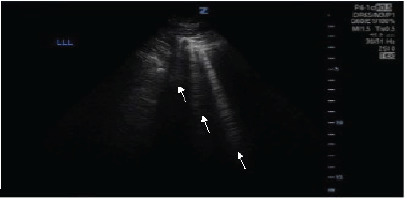
Initial chest radiograph upon emergency department arrival (left) and 15 hours after presentation (right) of a patient with chloramine exposure.

On arrival, vital signs were significant for hypoxia to 78% on room air, tachypnea of 32 breaths per minute, and tachycardia of 112 beats per minute. On exam, the patient was speaking in short sentences with diffuse wheezing bilaterally. Initial chest radiograph showed hazy and nodular opacities throughout both lungs (Image 1). He was initially given three nebulizer treatments of 2.5 mg albuterol and 0.5 mg ipratropium, 60 mg of prednisolone, 1000 mL normal saline, and 2 mg intravenous midazolam for agitation. Despite initial treatment, the patient’s tachypnea continued to worsen to greater than 40 breaths per minute, and he was placed on NIPPV at 10 cm of water (cmH_2_O)/5 cmH_2_O, fraction of inspired oxygen (FiO_2_) 25%. He was then transferred to the pediatric intensive care unit.

Repeat chest radiograph showed worsening opacities, requiring an increase in NIPPV settings to 16 cmH_2_O/8 cmH_2_O, FiO_2_ 30%, along with treatment with 114 mg methylprednisolone (2 mg/kg), continuous albuterol nebulization, and one dose of aerosolized 4.2% sodium bicarbonate (Image 1). The patient was weaned off NIPPV and albuterol nebulization after three days with improvement in his oxygenation and tachypnea. He was ultimately discharged on hospital day four.

### Case 2: Chlorine

A 58-year-old male with a history of asthma and uncontrolled diabetes presented to the ED with difficulty breathing after mixing sodium hypochlorite (~1–5%, exact concentration unknown) and hydrochloric acid (31.45%) in an enclosed bathroom while attempting to clean mold in his shower. Within minutes of the exposure, the patient developed dyspnea with no improvement after self-treatment with ipratropium bromide.

**Image 2:**
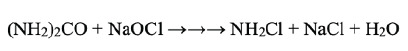
Left lower lung ultrasound with at least three B-lines present (arrows) in a 58-year-old male with chlorine exposure.

On arrival 45 minutes after the onset of symptoms, vital signs were the following: blood pressure 174/114 mm Hg, heart rate 125 beats per minute, respiratory rate 32 breaths per minute, and oxygen saturation of 85% on room air. Oxygen saturation improved to 98% with 15 L/minute oxygen via a non-rebreather mask with limited improvement in tachypnea. Pertinent findings on physical exam were diffuse wheezing in all lung fields. Point-of-care ultrasound (POCUS) revealed greater than three B-lines throughout the bilateral lower lung fields, sparing the upper and middle lung fields (Image 2).

Minimal improvement was found after treatment with nebulized albuterol/ipratropium and intravenous methylprednisolone, with persistent hypertension (205/104 mm Hg) and tachypnea to greater than 30 breaths per minite. Non-invasive positive pressure ventilation was initiated at 10 cmH_2_O/5 cmH_2_O, FiO_2_ 30%, respiratory rate of 16 breaths/minute with immediate relief and improved vital signs (blood pressure 160/98 mm Hg, heart rate 125 beats per minute, and respiratory rate 16 breaths per minute). Non-invasive positive pressure ventilation was implemented for 2.5 hours before transitioning to nasal cannula 2 liters. Repeat POCUS demonstrated resolving B-lines after NIPPV initiation. The patient ultimately was recommended admission to continue monitoring for delayed pulmonary edema but left against medical advice. Vital signs normalized prior to leaving seven hours after initial presentation and with follow-up 24 hours later demonstrating no symptoms or difficulty breathing.

## DISCUSSION

Chloramine, which includes monochloramine, dichloramine, and trichloramine, is produced from a combination of free chlorine and nitrogen-containing compounds. The aqueous solubility of the chloramines decreases as the size of the compound increases. Monochloramine (51 grams/mole [g/mol]) is easily soluble and stable in an aqueous solution.^8^ Dichloramine (85.92 g/mol) is also soluble but easily degrades through hydrolysis and base catalyzed reactions.^9^ Lastly, trichloramine (120.365 g/mol) is the least soluble in aqueous solutions and has a high vapor pressure, making inhalation injury more likely for trichloramine compared to mono- and dichloramine.^8^ In the first case, urine mixed with bleach produced chloramine through multiple possible chemical reactions. The most common includes ammonia in urine reacting with the sodium hypochlorite in bleach to produce chloramine gas and sodium hydroxide. The presence of urea can also form chlorurea, which through further chemical reactions will produce chloramine (Figure).

Urine as the source of nitrogen for the formation of chloramine exposure has been previously reported at an outbreak from an indoor swimming pool.^10^ However, adverse health effects from directly urinating in household bleach have not been noted in the literature. While pneumonitis may initially develop, persistent and worsening symptoms and imaging findings of pulmonary edema are consistent with ARDS.

Chlorine gas typically accumulates in low-lying areas since it is heavier than air with a molecular weight of 70.9 g/mol.^11^ Traditionally most injury is at the level of the larynx and segmental bronchi, as approximately 95% of chlorine gas is scrubbed by the upper respiratory tract.^11^,^12^ However, significant exposures appear to facilitate deeper penetration to the alveoli, leading to pneumonitis and, if untreated, severe ARDS. Our second case, with overt lower lung injury patterns on ultrasonography accompanied by significant hypoxemia, supports the finding that chlorine gas is associated with a predominate lower lung injury pattern on initial lung POCUS.

Initial treatment for both chloramine and chlorine includes aerosolized beta-2 agonists, aerosolized anticholinergics, and steroids to improve bronchospasm that can develop. Aerosolized sodium bicarbonate can also be used as an adjunct therapy if given early in disease course for acid neutralization. While sodium bicarbonate has shown to be beneficial in the acute phase of chlorine gas toxicity, the benefit in chloramine toxicity is not as well known. However, it is still a safe treatment option.^13^,^14^

## CONCLUSION

These cases support several essential findings for emergency physicians. First, noninvasive positive pressure ventilation can effectively assist in the treatment of chlorine gas pneumonitis and chloramine ARDS, in this case facilitating pediatric respiratory support without requiring intubation. Noninvasive positive pressure ventilation should represent an essential first-line treatment for emergency physicians treating patients with significant tachypnea or hypoxemia secondary to chlorine and chloramine gases. Second, our findings suggest that initial ultrasonographic response to NIPPV may differentiate irritant gas pneumonitis from ARDS, with reversible pneumonitis carrying resolving B-lines after NIPPV initiation while ARDS may have persistent and diffuse B-lines. Further research is required for initial and continued ultrasonographic monitoring of patients with exposure to chlorine gas agents.

## Figures and Tables

**Figure:**
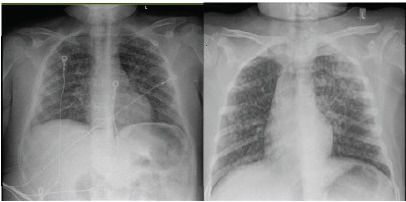
Simplified reaction of urea with sodium hypochlorite. Abbreviations: H2O, water; (NH_2_)_2_CO, urea; NaOCl, sodium hypochlorite; NH_2_Cl, monochloramine; NaCl, sodium chloride.
